# Effect of Environmental pH on Mineralization of Anaerobic Iron-Oxidizing Bacteria

**DOI:** 10.3389/fmicb.2022.885098

**Published:** 2022-05-12

**Authors:** Na Jiang, Yiqing Feng, Qiang Huang, Xiaoling Liu, Yuan Guo, Zhen Yang, Chao Peng, Shun Li, Likai Hao

**Affiliations:** ^1^State Key Laboratory of Environmental Geochemistry, Institute of Geochemistry, Chinese Academy of Sciences, Guiyang, China; ^2^Institute of Geochemistry, University of Chinese Academy of Sciences, Beijing, China; ^3^Minzu Normal University of Xingyi, Xingyi, China; ^4^College of Urban and Environmental Science, Peking University, Beijing, China; ^5^College of Life Sciences, China West Normal University, Nanchong, China; ^6^Ningbo Urban Environment Observation and Research Station, Chinese Academy of Sciences, Ningbo, China; ^7^Chinese Academy of Sciences (CAS) Center for Excellence in Quaternary Science and Global Change, Xi'an, China

**Keywords:** iron oxidizers, goethite, bio-oxidation, FIB–SEM, TEM

## Abstract

Freshwater lakes are often polluted with various heavy metals in the Anthropocene. The iron-oxidizing microorganisms and their mineralized products can coprecipitate with many heavy metals, including Al, Zn, Cu, Cd, and Cr. As such, microbial iron oxidation can exert a profound impact on environmental remediation. The environmental pH is a key determinant regulating microbial growth and mineralization and then influences the structure of the final mineralized products of anaerobic iron-oxidizing bacteria. Freshwater lakes, in general, are neutral-pH environments. Understanding the effects of varying pH on the mineralization of iron-oxidizing bacteria under neutrophilic conditions could aid in finding out the optimal pH values that promote the coprecipitation of heavy metals. Here, two typical neutrophilic Fe(II)-oxidizing bacteria, the nitrate-reducing *Acidovorax* sp. strain BoFeN1 and the anoxygenic phototrophic *Rhodobacter ferrooxidans* strain SW2, were selected for studying how their growth and mineralization response to slight changes in circumneutral pH. By employing focused ion beam/scanning electron microscopy (FIB–SEM) and transmission electron microscopy (TEM), we examined the interplay between pH changes and anaerobic iron-oxidizing bacteria and observed that pH can significantly impact the microbial mineralization process and vice versa. Further, pH-dependent changes in the structure of mineralized products of bacterial iron oxidation were observed. Our study could provide mechanical insights into how to manipulate microbial iron oxidation for facilitating remediation of heavy metals in the environment.

## Introduction

Anaerobic iron-oxidizing bacteria can catalyze the oxidation of ferrous iron [Fe(II)] to ferric iron [Fe(III)] minerals under anoxic conditions (Hedrich et al., 2011; Bryce et al., 2018). The bacteria and their Fe(III) mineral precipitations can adsorb and coprecipitate not only the nutrients, such as phosphorus and carbon (Schmid et al., 2014; Kappler et al., 2021), but also the heavy metals, including Al, Zn, Cu, Cd, Cr, Mn, and Co (Abramov et al., 2020; Aziz et al., 2020). Microbially-mediated anaerobic iron oxidation has important geological significance (Xiu et al., 2019). As an example, the formation of Archean–Paleoproterozoic banded iron formations (BIFs) was thought to be related to anaerobic iron-oxidizing bacteria (Konhauser et al., 2007). Environmental pH is the main factor affecting the growth of microorganisms (Ghosh et al., 2016) by modulating enzyme activity, protein conformation, and functional group activity (Hoštacká et a1., 2010; Aryal and Liakopoulou-Kyriakides, 2014). Even a slight change in environmental pH can exert a significant impact on their growth (Patil et al., 2011). In addition, environmental pH can directly affect the precipitation of iron ions in the process of iron oxidation. It can also influence the types of Fe(II) species formed and enhance their reactivity when pH increases (Huang et al., 2021). For example, *Rhodopseudomonas palustris* TIE-1 was shown to form different mineralized products under distinct pH pressures (Jiao and Newman, 2007), and poorly crystalline Fe(III) oxyhydroxides and goethite can be formed at low pH, while magnetite was observed at high pH (Bryce et al., 2018). The strain *Acidovorax* sp. BoFeN1 can also form different mineralized products under pH 7.7 and pH 6.3 (Larese-Casanova et al., 2010).

As outlined above, environmental pH is a strong regulator for bio-oxidation of ferrous iron (Fan et al., 2019) and Fe(III) mineral precipitations. The spherical surface complexes formed by microbial mineralization can show a high affinity for heavy metals at neutral pH (Tong et al., 2019). At present, microscopic tools, including scanning electron microscopy (SEM) (Schädler et al., 2009), scanning transmission (soft) X-ray microscopy (STXM) (Pantke et al., 2012), and confocal laser scanning microscopy (CLSM) (Hao et al., 2013), have been well employed in studying iron-oxidizing bacteria on a larger pH scale. In this study, we combined transmission electron microscope (TEM), X-ray diffraction (XRD), and focused ion beam (FIB)/scanning electron microscopy (SEM) to examine the microscopic relationship between pH and anaerobic iron-oxidizing bacteria during the iron oxidation process and further provide a theoretical basis for future applications of anaerobic iron-oxidizing bacteria in the remediation of environmental heavy metal pollution.

## Materials and Methods

### Bacterial Strains and Cultivation Conditions

*Acidovorax* sp. BoFeN1 (Kappler et al., 2005) and *Rhodobacter ferrooxidans* SW2 (Ehrenreich and Widdel, 1994) were used as model strains to analyze the iron oxidation under different pH pressures. *Acidovorax* sp. strain BoFeN1 is a nitrate-reducing bacterium that can induce Fe(II) oxidation (Klueglein et al., 2013), isolated from Lake Constance littoral sediments. *Rhodobacter ferrooxidans* SW2 is a purple non-sulfur bacterium that can oxidize Fe(II) phototrophically (Hohmann et al., 2010; Pereira et al., 2017). The low-phosphate anoxic mineral medium (Widdel, 1980; Parkes et al., 2010) was prepared for the growth of all strains, containing 0.3 g·L^−1^ of NH_4_Cl, 0.14 g·L^−1^ of KH_2_PO_4_, 0.5 g·L^−1^ of MgSO_4_·7H_2_O, 0.1 g·L^−1^ of CaCl_2_·2H_2_O, and 0.2 g·L^−1^ of NaCl. The medium was autoclaved (121°C, 1.01 × 10^5^ Pa, 30 min) and cooled down and then was supplied with 1.85 g·L^−1^ of NaHCO_3_, 1 mL·L^−1^ of trace element solution, 1 mL·L^−1^ of selenite–tungstate solution, and 1 mL·L^−1^ of vitamin solution (Kappler et al., 2005). Next, the medium was dispensed into sterile penicillin bottles and the headspace was flushed with N_2_/CO_2_ (*V*/*V*, 80%/20%). To further evaluate the effects of environmental pH on mineralization, the pH of the medium was adjusted to the required values (6.8, 6.9, 7.0, 7.1, and 7.2) by adding Na_2_CO_3_ or HCl from stock sterile solutions. *Acidovorax* sp. strain BoFeN1 was cultivated in 10 mL anoxic medium at different pHs, containing 10 mM of NaNO_3_, 10 mM of FeCl_2_, and 5 mM of acetate. The inoculation was 5%, and the strain was incubated at 28°C in the dark for 16 days. The pre-culture of *Acidovorax* sp. strain BoFeN1 has been cultivated for 192 h to make it growing to the log phase. *Rhodobacter ferrooxidans* strain SW2 was cultivated in 10 mL anoxic medium at indicated pHs, with 10 mM of FeCl_2_ as the electron donor. The inoculation was 5%, and the strain was incubated at 20°C under incandescent light for 31 days. The pre-culture of *Rhodobacter ferrooxidans* strain SW2 has been cultivated for 480 h to enable it growing to the log phase. All treatments were performed in independent triplicates.

### Microbial Growth and Mineralization Experiments

#### Cell Growth

The OD_600_ was measured to monitor the cell growth (Suter et al., 1988). Two milliliters of cultivated samples was centrifuged at 12,000 r·min^−1^ for 10 min, 1.5 mL supernatant was discarded, and then, 0.45 mL oxalate solution and 0.05 mL 100 mmol· L^−1^ of ferrous ethylenediammonium sulfate solution were added. After fully mixing, 200 μL of mixtures was measured by a 96-well microplate reader with a maximum absorbance at 600 nm (Multiskan FC, Thermo Scientific). The ferrozine assay (Stookey, 1970) was used to determine the changes in iron ion concentration in culture bottles. In an anoxic glove box (Vinyl Anaerobic Chambers), a 30 μL sample was mixed with 270 μL 1 M of HCl. For total Fe, 20 μL of test solution was added with 80 μL 100 g· L^−1^ of HAHCl. For Fe(II), a 20 μL test solution was added with 80 μL 1 M of HCl. After incubation in dark for 30 min, 100 μL 1 g· L^−1^ of ferrozine solution was added. After 5-min incubation, the sample was measured by a 96-well microplate reader with a maximum absorbance at 560 nm. With respect to the mineralization experiment, 2 mL of cultivated samples was taken with sterile syringes at indicated time intervals in the glove box (mixed thoroughly), and the pH value of the sample was measured by an electronic pH meter at room temperature.

#### Mineral Characterization by XRD, Raman

At the logarithmic to stable phase, 50 mL of cultivated samples was collected and washed with deoxidized distilled water for three times, then filtered with a 0.22-μm pore size filter, and dried in an anoxic glove box. XRD spectra were obtained by an X-ray powder diffractometer (Empyrean, Netherlands) using Cu-K α radiation operating at 40 kV and 40 mA. The Raman spectra were acquired by microscopic confocal laser Raman spectroscopy (Renishaw inVia Reflex, UK) with 50 × objective magnification, 1,800 gr· mm^−1^ grating, 1.5 μm focus spot, and 325 nm excitation wavelength.

#### Morphologic Observation

To obtain morphologic information on the two microbial strains during iron oxidation, on the last day of the incubation experiment aliquots of 2 μL were collected, deposited on the 300-mesh carbon-coated Cu grid, and air-dried in an anoxic glove box. Bright-field TEM images were acquired using TEM (Tecnai G^2^ F20 S-TWIN, USA) operated at 200 kV of accelerated voltage.

Similarly, for FIB–SEM 3D reconstruction, resin block is prepared as described elsewhere (Schmid et al., 2014). In brief, samples were concentrated by low-speed centrifugation (5,000 r·min^−1^, 10 min) of cell suspension from a stable phase. Cellulose capillary filled with concentrated samples was packed into specimen carrier to prepare for the next step, filled with 1-hexadecene. The specimen carrier was high-pressure frozen using a Leica EM HPM100, followed by freeze substitution (Leica EM AFS2) with 1.25% glutaraldehyde in acetone. The temperature was stepwise increased from −90 to −60°C, then to −40°C, and finally to 0°C. After substitution, samples were washed with acetone and Epon infiltrated stepwise and then embedded in fresh Epon for 2 days at 60°C.

Using a focused ion beam to cut one piece of Epon block, the cut Epon block is close to the area of interest, glued on SEM stub with silver paint and transferred to FEI Scios™ DualBeam system. With a protective layer of Pt, the block was coarse-milled and fine-milled with Ga^2+^ ion at acceleration voltage of 30 kV. Subsequently, the slice and view software was started, and slice thickness was set to 20 nm, ion current was 0.6 pA-65 nA, image pixels was ≤ 5.0 nm@30 kV and accelerating voltage was 0.5 kV-30 kV. For 3D construction, a series of sliced images were combined into a single 3D volume using Amira.

## Results and Discussion

### Microbial Fe(II) Oxidation

**Figure 1:**
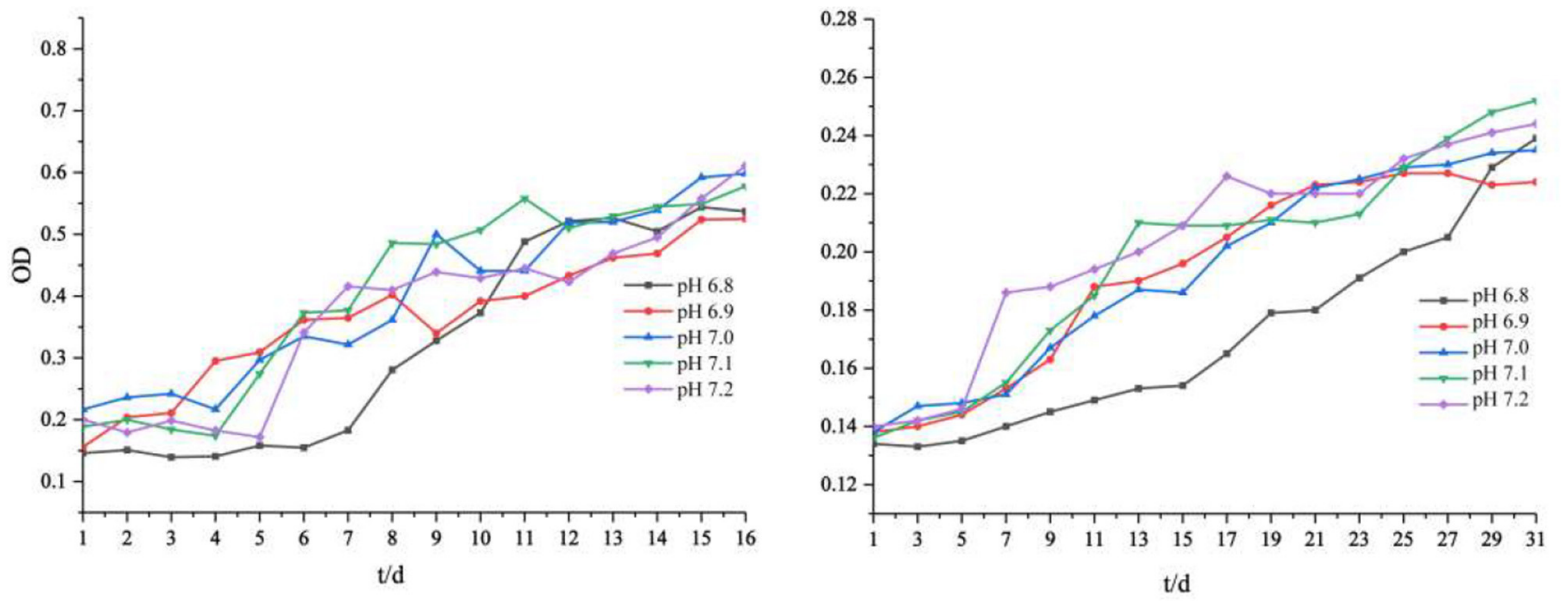
Cell growth (OD_600_) of *Acidovorax* sp. strain BoFeN1 **(left)** and *Rhodobacter ferrooxidans* strain SW2 **(right)** with different initial pHs.

The slight fluctuation of environmental pH affected the growth rate and biomass of iron-oxidizing bacteria (Figure 1). Our results showed that lower pH values of the medium led to lower final biomass of cells. The total biomass of the two strains was different but showed the same trend. The cell biomass at pH 7.1 and 7.2 was slightly higher than that at pH 6.8 and 6.9. In addition, we observed that the lower the initial pH value of the medium, the longer the lag phase of *Acidovorax* sp. strain BoFeN1. When the initial pH of the medium solution was 6.9–7.2, the average lag phase of *Acidovorax* sp. strain BoFeN1 was about 4 days. When the initial pH dropped to 6.8, the lag phase was up to 6 days. This is because environmental pH is the main factor affecting the growth of all microorganisms (Ghosh et al., 2016). Each bacterium has a pH range suitable for its own growth. The farther the pH of the environment is from the optimal pH, the lower the electrocatalytic activity and efficiency of the microbial biofilm, and the more negative influence affected the growth of the microorganism (Patil et al., 2011). Compared with *Acidovorax* sp. strain BoFeN1, *Rhodobacter ferrooxidans* strain SW2 grew much more slowly. Medium with an initial pH of 6.8 also significantly slowed the growth of *Rhodobacter ferrooxidans* strain SW2. It is known that the adsorption of metal ions by microorganisms depends largely on their growth stage, and the adsorption amount is highest in the logarithmic phase (Fan et al., 2014). Hence, pH 6.8 may significantly affect the adsorption and coprecipitation behaviors of those two strains.

**Figure 2:**
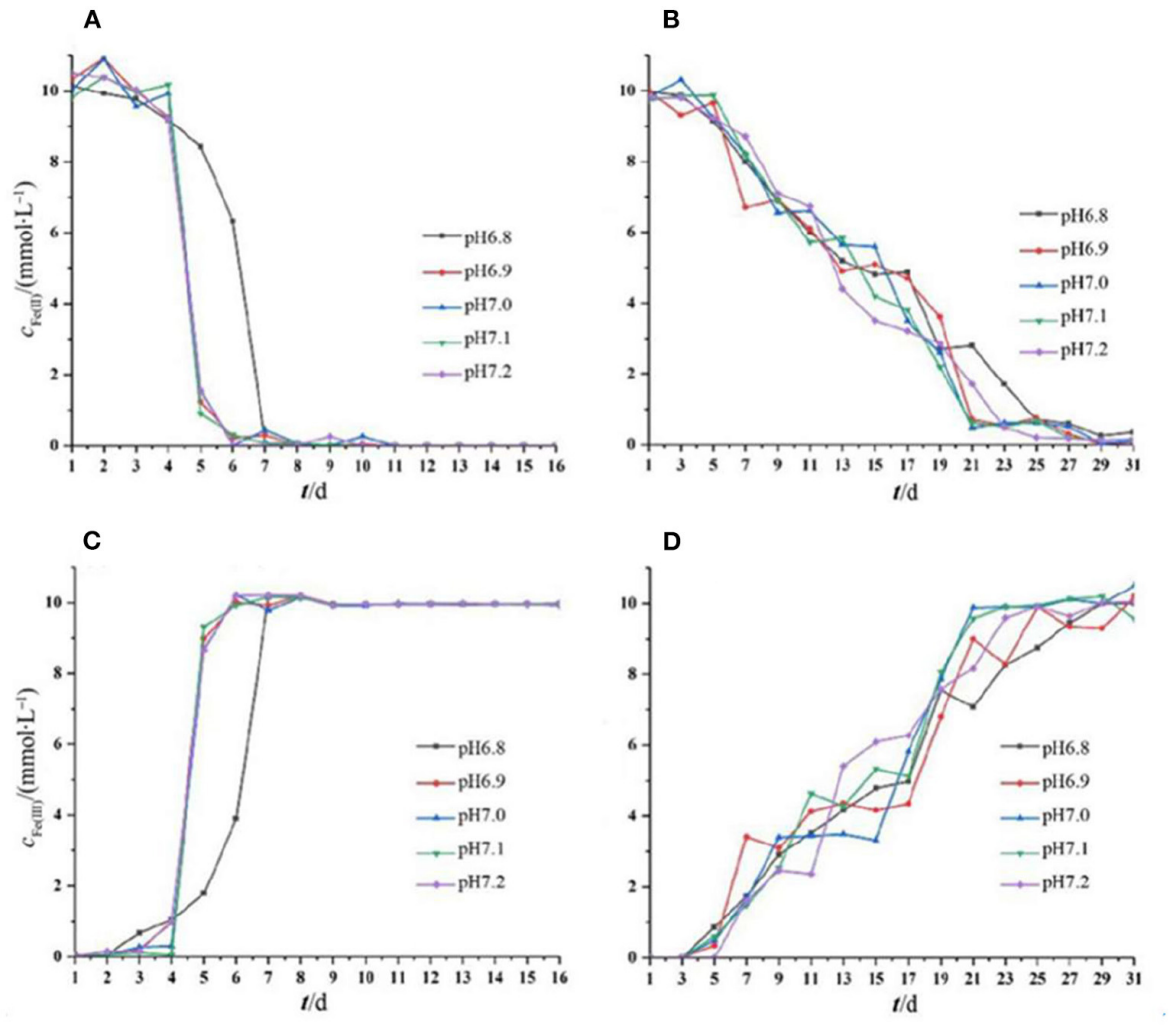
Changes in Fe(II) and Fe(III) concentration throughout the period of cell growth under different pH pressures. Each experiment was run in triplicate cultures. Decreased Fe(II) concentration of strain BoFeN1 **(A)** and strain SW2 **(B)**, and increased Fe(III) concentration of strain BoFeN1 **(C)** and strain SW2 **(D)**.

The oxidation of iron by the two strains supported the cell growth. For *Acidovorax* sp. strain BoFeN1, Fe^2+^ and Fe^3+^ did not change in the initial 4 days, and after that, Fe^2+^ was completely oxidized within 2 days. Fe^2+^ was consumed rapidly, as it can be enzymatically oxidized by the bacterium and abiotically oxidized by NO_2_ and NO produced from nitrite reduction (Klueglein and Kappler, 2013). Very recently, it was reported that biological Fe^2+^ oxidation was dominant in the iron oxidation process (Dopffel et al., 2021). It was suggested that in the oxidation of iron by *Acidovorax* sp. strain BoFeN1, the microorganisms themselves were mainly responsible for the complete oxidation of Fe^2+^ within 2 days. The curve of Fe(III) production of *Rhodobacter ferrooxidans* strain SW2 was highly consistent with its growth curve (Figure 2). There were almost no changes in Fe^2+^ and Fe^3+^ in the initial 5 days, and then, Fe^2+^ was gradually oxidized to Fe^3+^. In general, except for that the iron oxidation of *Acidovorax* sp. strain BoFeN1 was inhibited at pH 6.8, there was no significant difference in the rates of Fe^2+^ oxidation by the two iron-oxidizing bacteria cultivated under different initial pH pressures.

### Changes in pH

**Figure 3:**
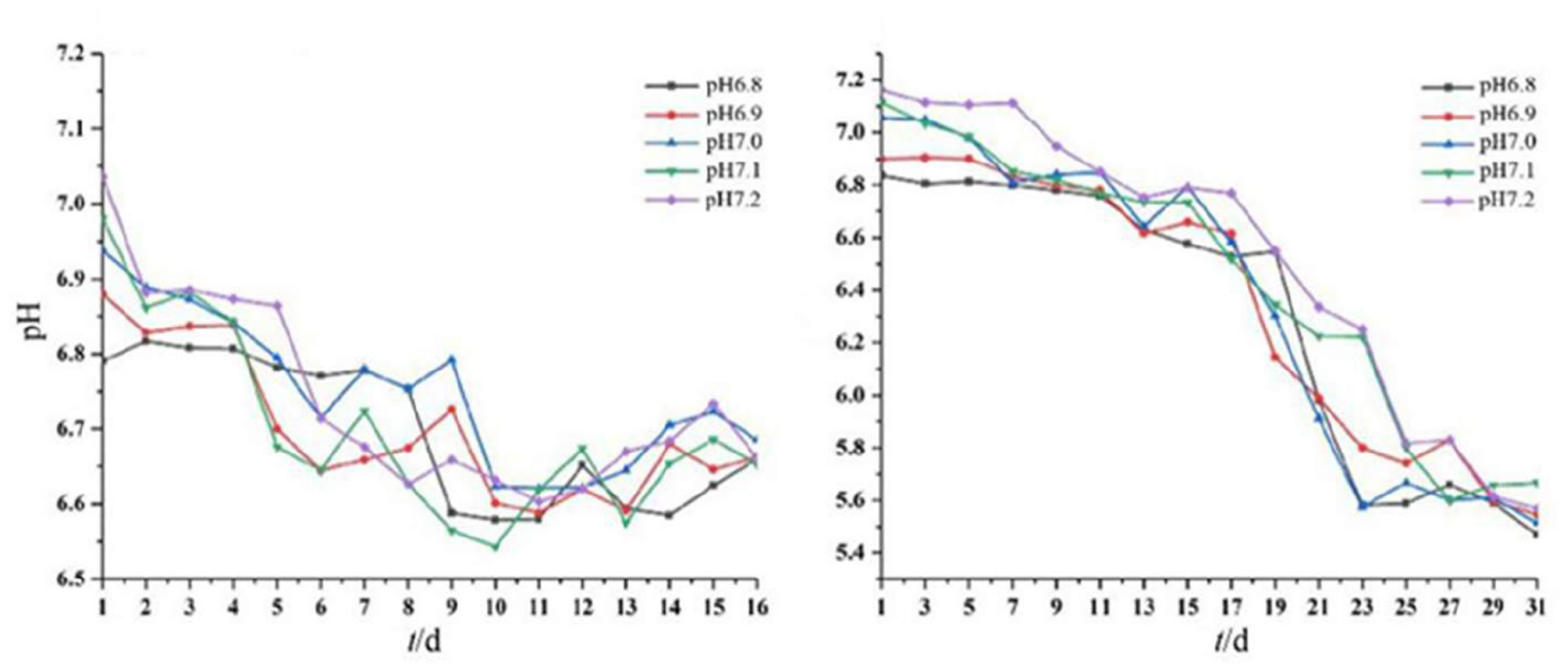
Dynamic changes in pH of *Acidovorax* sp. strain BoFeN1 over 16 days **(left)** and *Rhodobacter ferrooxidans* strain over 31 days **(right)**.

The pH of the medium decreased gradually with the growth of the two strains (Figure 3). The solution pH of *Acidovorax* sp. strain BoFeN1 decreased from the initial range of 6.8–7.2 to the range of 6.5–6.7. This is likely because Fe^3+^ formed during Fe^2+^ oxidation by the two iron-oxidizing bacteria could combine with OH^−^ in the solution (10${\text{Fe}}^{2+}+2NO_{3}^{-}+24H_{2}O\to 10Fe{\left(OH\right)}_{3}+N_{2}+18H^{+}$) (Klueglein et al., 2013). The pH of *Rhodobacter ferrooxidans* strain SW2 decreased to a larger extent than *Acidovorax* sp. strain BoFeN1. The solution pH of *Rhodobacter ferrooxidans* strain SW2 decreased from the initial range of 6.8–7.2 to the range of 5.5–5.8. First, the oxidized Fe^3+^ is also bound to OH^−^ (${\text{HCO}}_{3}^{-}+4Fe^{2+}+10H_{2}O\to CH_{2}O+4Fe{\left(OH\right)}_{3}+7H^{+}$) (Pereira et al., 2017), resulting in a decrease in the pH value. Second, C-type cytochrome foxE was found to be involved in the iron oxidation pathway of *Rhodobacter ferrooxidans* strain SW2, resulting in a better diffusion of the generated ferric hydroxide (Croal et al., 2007). To prevent ferric compounds from crusting on the cell surface and thus affecting the normal growth of microorganisms, both strains of bacteria have evolved metabolic strategies to reduce the microenvironmental pH around the cells. For example, proton motive force (PMF) produced a slightly acidic microenvironment so that Fe(III) was dissolved and was activated near the cells, thus achieving the purpose of migration and transfer of ferric ions (Johnson et al., 2007; Schädler et al., 2009).

These results indicated that the environmental pH affected the growth of the two strains and vice versa. This drop in pH has also been observed by other studies. As an example, the pH of the environment around the iron-oxidizing bacterium *Thiodictyon* sp. strain F4 growing in a solid medium was lower than cells and distant microenvironments, as evidenced by microelectrode measurement and chemically labeled confocal laser scanning microscopy (Hegler et al., 2010). Another example is that microbial iron oxidation in pig manures induced changes in environmental pH, decreasing from 6.34 to 2.4 within 12 days of incubation (Wei et al., 2020). Together, data showed that the metabolism of iron-oxidizing bacteria can acidify the microenvironment deeply around or between cells (Kappler and Newman, 2004). In addition, the solubility of iron ions is higher under acidic conditions, so the acidification of *Acidovorax* sp. strain BoFeN1 and *Rhodobacter ferrooxidans* strain SW2 may affect the degree of crystallization of microbial mineralization products.

Environmental pH is an important variable controlling the adsorption of metal ions to microorganisms (Hua et al., 2019). The optimum pH range for the adsorption of many heavy metals, such as Pb(II), Cu (II), Ni(II), Zn(II), Cd(II), and Cr(III), is 5–6 (Kumari et al., 2017; Zhang et al., 2017; Xu et al., 2020). *Rhodobacter ferrooxidans* strain SW2 and *Acidovorax* sp. strain BoFeN1 can decrease the environmental pH to 5–6 during iron oxidation; thus, these two strains have an important application prospect in the bioremediation of heavy metal ions.

### Spectroscopic Characteristics of Mineralized Products

#### Mineralogical Analysis

**Figure 4:**
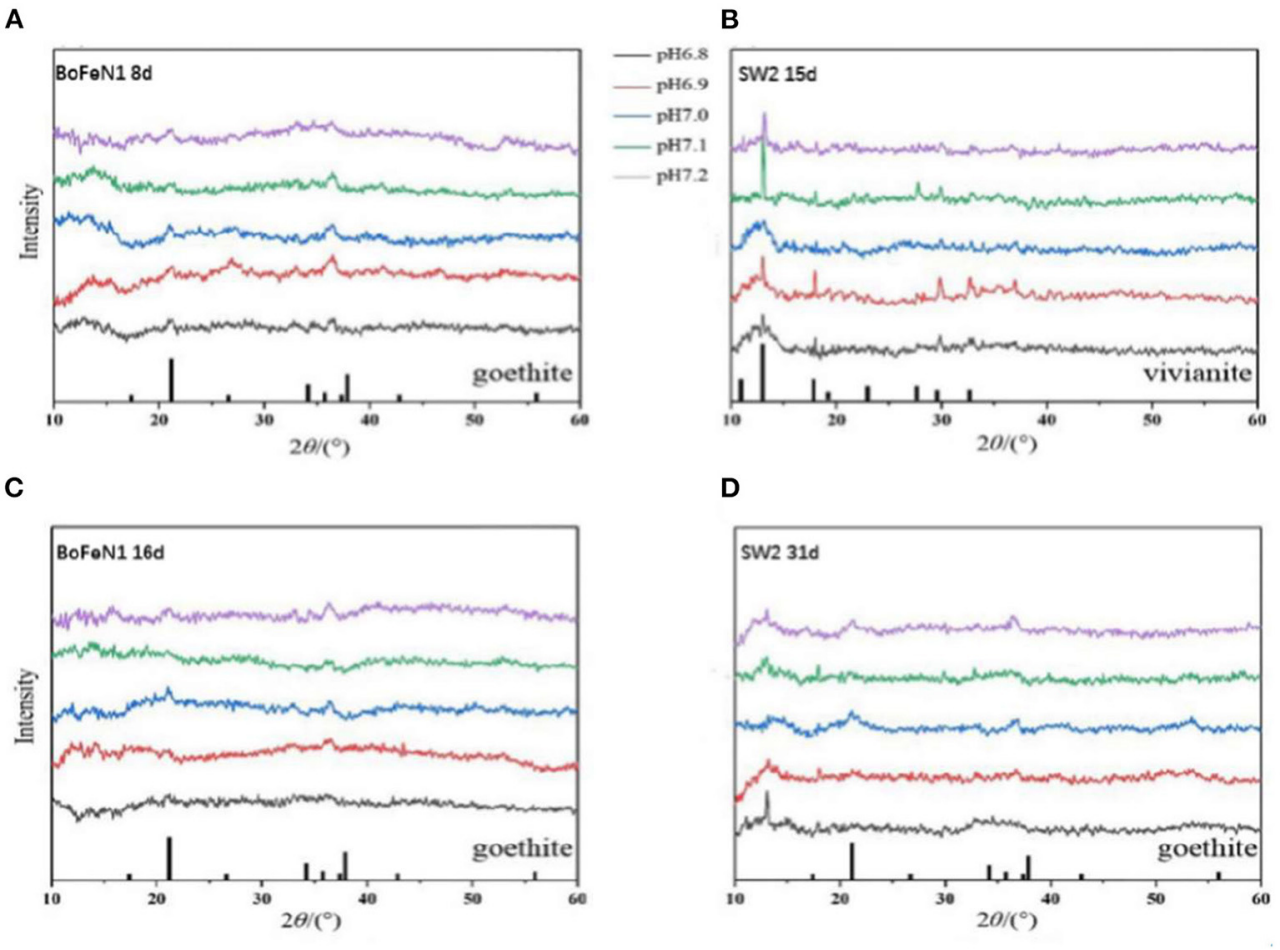
X-ray diffractograms of precipitates collected from **(A)** 8 days of *Acidovorax* sp. strain BoFeN1; **(B)** 15 days of *Rhodobacter ferrooxidans* strain SW2; **(C)** 16 days of *Acidovorax* sp. strain BoFeN1; and **(D)** 31 days of *Rhodobacter ferrooxidans* strain SW2. The bottom of **(A,C,D)** marks the diffraction peak of goethite, and the bottom of **(B)** marks the diffraction peak of vivianite.

To further study the mineralization of anaerobic iron-oxidizing bacteria, we used XRD to examine the powder samples from the middle and last stages of incubation with *Acidovorax* sp. strain BoFeN1 and *Rhodobacter ferrooxidans* strain SW2. As shown in Figure 4, the XRD patterns of *Acidovorax* sp. strain BoFeN1 in the middle and at the end of the experiment were consistent with the diffraction characteristics of goethite. It shows that the lower initial pH of the medium can decrease the crystallinity of the sample.

In the middle stage of the experiment, the XRD pattern of *Rhodobacter ferrooxidans* strain SW2 was in agreement with the diffraction characteristics of vivianite (Figure 4B), while at the end of the experiment, the vivianite gradually disappeared, replacing by goethite with low crystallinity (Figure 4D). Previous experiments indicate that the phosphate and carbonate contained in the medium can form soluble complexes and precipitates with ferric ions, which can destroy the crystallization and affect the mineral properties of ferric oxides (Larese-Casanova et al., 2010). At the same time, extracellular polymer (EPS) production was shown to inhibit the growth of mineral crystals (Klueglein et al., 2013). That is why the final mineralized product of *Rhodobacter ferrooxidans* strain SW2 is goethite with low crystallinity. The structure of the final mineralized products of the two strains was nearly identical, and the influence of environmental pH on them was similar, that is, the lower the initial pH value of the medium, the worse the crystallinity, because higher pH would increase the adsorption and reactivity of microorganisms to Fe^2+^ (Huang et al., 2021).

As for vivianite, because the medium could form white precipitation with FeCl_2_, vivianite existed at the initial stage of culture of both strains. Note that there was no trace amount of vivianite in the middle and late spectrograms of *Acidovorax* sp. strain BoFeN1, which indicated that the ability of *Acidovorax* sp. strain BoFeN1 for the utilization of ferric ions in Fe(II) minerals with certain crystallinity was stronger than that of *Rhodobacter ferrooxidans* strain SW2 (Miot et al., 2009c). *Acidovorax* sp. strain BoFeN1 could also proceed abiotically *via* the secretion of reactive nitrite outside the cell (Miot et al., 2009a).

#### Mineral Morphology

**Figure 5:**
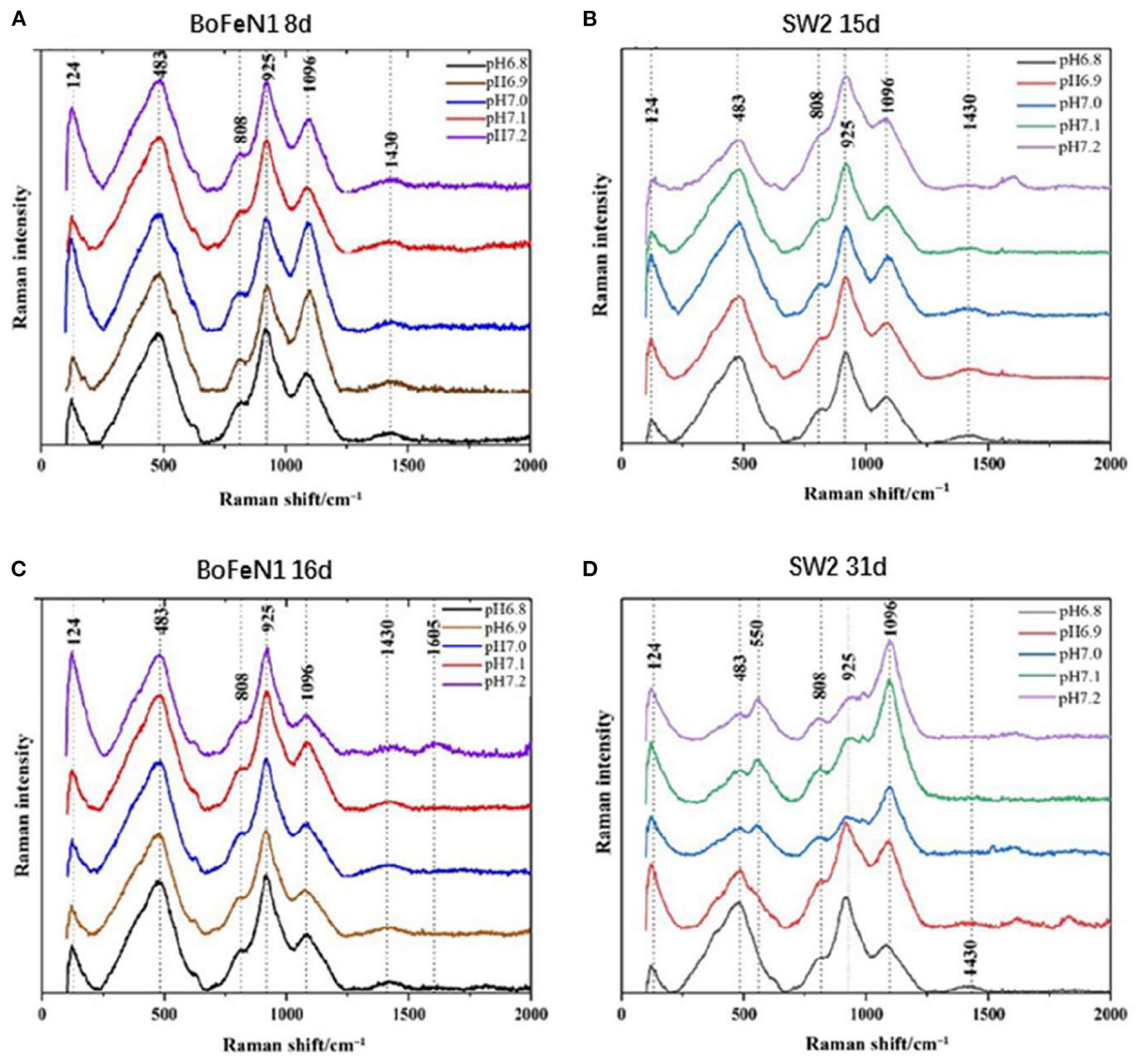
Raman spectra of precipitates collected from **(A)** 8 d of *Acidovorax* sp. strain BoFeN1; **(B)** 15 d of *Rhodobacter ferrooxidans* strain SW2; **(C)** 16 d of *Acidovorax* sp. strain BoFeN1; and **(D)** 31 d of *Rhodobacter ferrooxidans* strain SW2.

XRD is not qualified to analyze the poorly crystalline minerals (Huang et al., 2021), so Raman spectroscopy is used for analyzing chemical groups and mineralogy of mineralization. The information obtained from the Raman pattern (Figure 5) was consistent with the results of XRD. The products of *Acidovorax* sp. strain BoFeN1 in the middle and end of the experiment were almost the same. The product characterized by the characteristic peaks of 124 and 483 cm^−1^ was goethite (De Faria and Lopes, 2007), among which the characteristic peak of 1,096 cm^−1^ mainly represented the vibration of C–OH group (Laso-Pérez et al., 2018), 808 cm^−1^ represented the stretching vibration of O–P–O bond (Huayhongthong et al., 2019), and 1,430 cm^−1^ represented the deformation vibration of C–H which might be –CH_2_ and –CH_3_ on the cell membrane (Lorenz et al., 2017). In addition, the characteristic peak of 925 cm^−1^ might be formed by the deformation and vibration of C=C bond, which also contained the characteristic peak of P–OH in phosphate (920–970 cm^−1^) (Huayhongthong et al., 2019). It showed that the product of *Acidovorax* sp. strain BoFeN1 was goethite, which also contained some phosphate.

There were differences in the Raman spectra of *Rhodobacter ferrooxidans* strain SW2 samples between the middle and last stages of cultivation. However, the response of the samples under different initial medium pHs was not consistent. The Raman spectra of the samples at pH 7.0, 7.1, and 7.2 showed obvious peak changes in the middle and last stages. The characteristic peak of 925 cm^−1^ on 31 days was lower than the peak of that on 15 days, which indicated the decrease in Raman signal of vivianite. The increase in characteristic peak of 1,096 cm^−1^ represented the gradual secretion of lipids and polysaccharides by microorganisms. However, at pH 6.8 and pH 6.9, it remained unchanged, which indicated the low pH of the initial medium would affect the formation of mineral product.

### Micromorphological Characteristics of Iron-Oxidizing Bacteria

**Figure 6:**
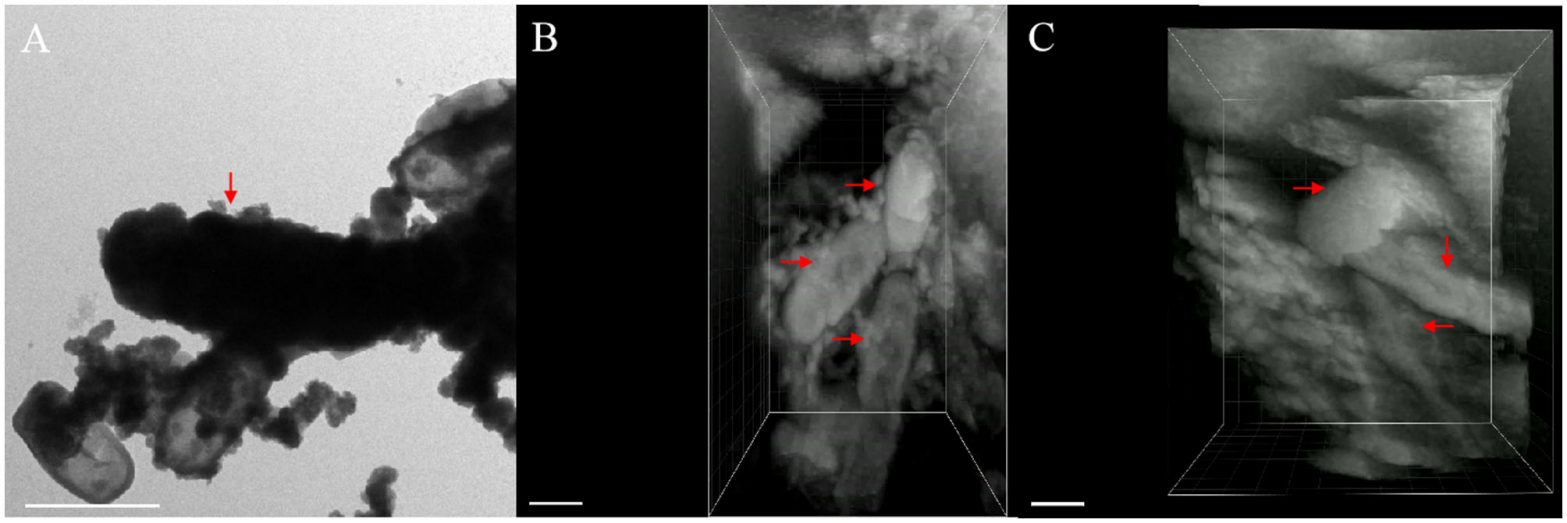
TEM images of cell mineralization oxidizing by *Acidovorax* sp. strain BoFeN1 **(A,B)** and *Rhodobacter ferrooxidans* strain SW2 **(C,D)**, scale bar = 1 μm.

Bright-field TEM images showed that the morphology of minerals on the cell surface of *Acidovorax* sp. strain BoFeN1 and *Rhodobacter ferrooxidans* strain SW2 was completely different. The cell surface of *Acidovorax* sp. strain BoFeN1 was mainly attached to the nano-sized mineral particles (Figure 6A), because there were preferential Fe-blinding and Fe-oxidizing sites on the plasma membrane (Miot et al., 2011). These particles grew with the oxidation process (Figure 6B), and finally, the whole cell surface was encrusted (Schädler et al., 2009). They were considered as amorphous iron phosphate precipitation (Miot et al., 2009b) and iron hydroxide (Bryce et al., 2018), which promoted the microbial transport, absorption, and utilization of iron, and were considered as intermediate products before goethite.

In contrast to *Acidovorax* sp. strain BoFeN1, cells of *Rhodobacter ferrooxidans* strain SW2 were encapsulated by filamentous substance but not crusted, showing a state of fibrosis (Figure 6C). In addition, a kind of nearly spherical mineralized substance of large diameter was observed on the surface of cells (Figure 6D). The surface layer of the spherical material was also fibrous; maybe the spherical substance had a structure folded by fibrous material. These fibers are mainly formed by lipids and polysaccharides secreted by cells (Gupta and Diwan, 2017), and then, nano-sized goethite precipitates these fibers as templates on the fibers to form fibrous mineralized products (Miot et al., 2009b).

According to TEM images, both strains had their own unique regulatory mechanism to migrate iron minerals, thereby preventing the cell crusting from affecting the growth. For example, EPS production was a strategy to decrease encrustation (Klueglein et al., 2013). However, our results showed that *Acidovorax* sp. strain BoFeN1 was encapsulated by a mineralized substance, while *Rhodobacter ferrooxidans* strain SW2 was not, indicating that *Rhodobacter ferrooxidans* strain SW2 had a stronger ability to transfer and regulate Fe^3+^ than *Acidovorax* sp. strain BoFeN1. From the change in the pH curve of samples, it could be seen that the pH of *Rhodobacter ferrooxidans* strain SW2 solution decreased much more than that of *Acidovorax* sp. strain BoFeN1. It showed that *Rhodobacter ferrooxidans* strain SW2 had a stronger ability to regulate the microenvironmental pH during iron oxidation. TEM and EDX were used to analyze *Rhodobacter ferrooxidans* strain SW2 and *Acidovorax* sp. strain BoFeN1 at the middle and end of the experiment under different pH conditions. By comparing TEM image and EDX information, it was found that different initial medium pHs (6.8–7.2) had little effect on microimaging of both strains.

### FIB–SEM 3D Reconstruction of *Acidovorax* sp. Strain BoFeN1

**Figure 7:**
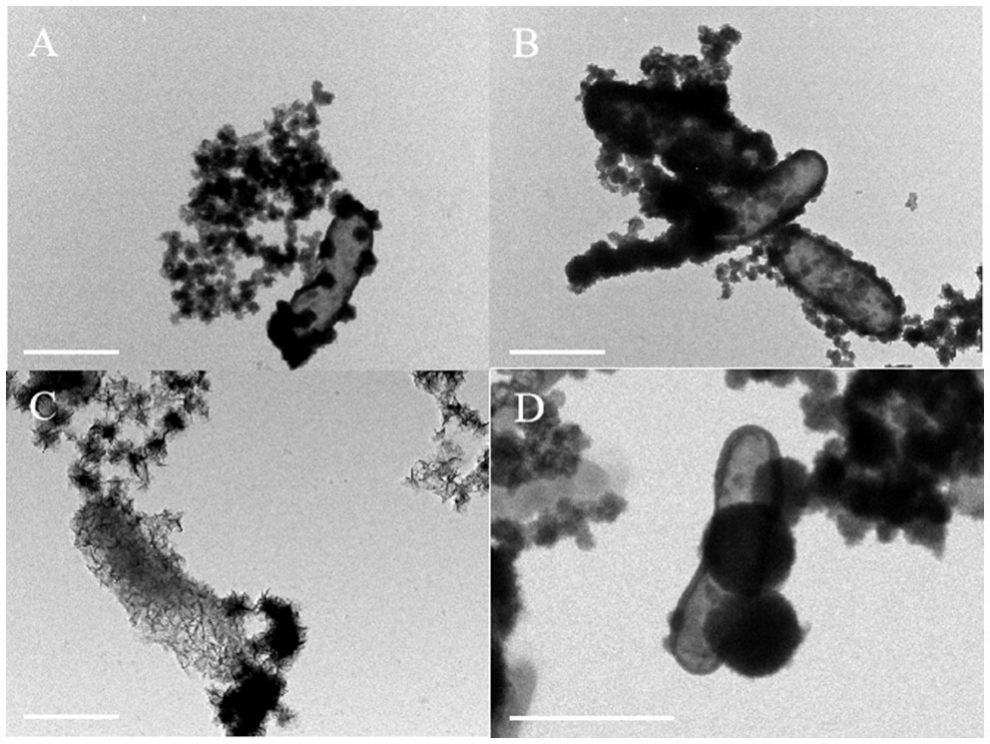
TEM image containing encrusted cell [**(A)**, red arrow] and different perspective images of FIB/SEM tomography **(B,C)** with fully and partially encrusted cells (red arrow), scale bar = 1 μm.

**Figure 8:**
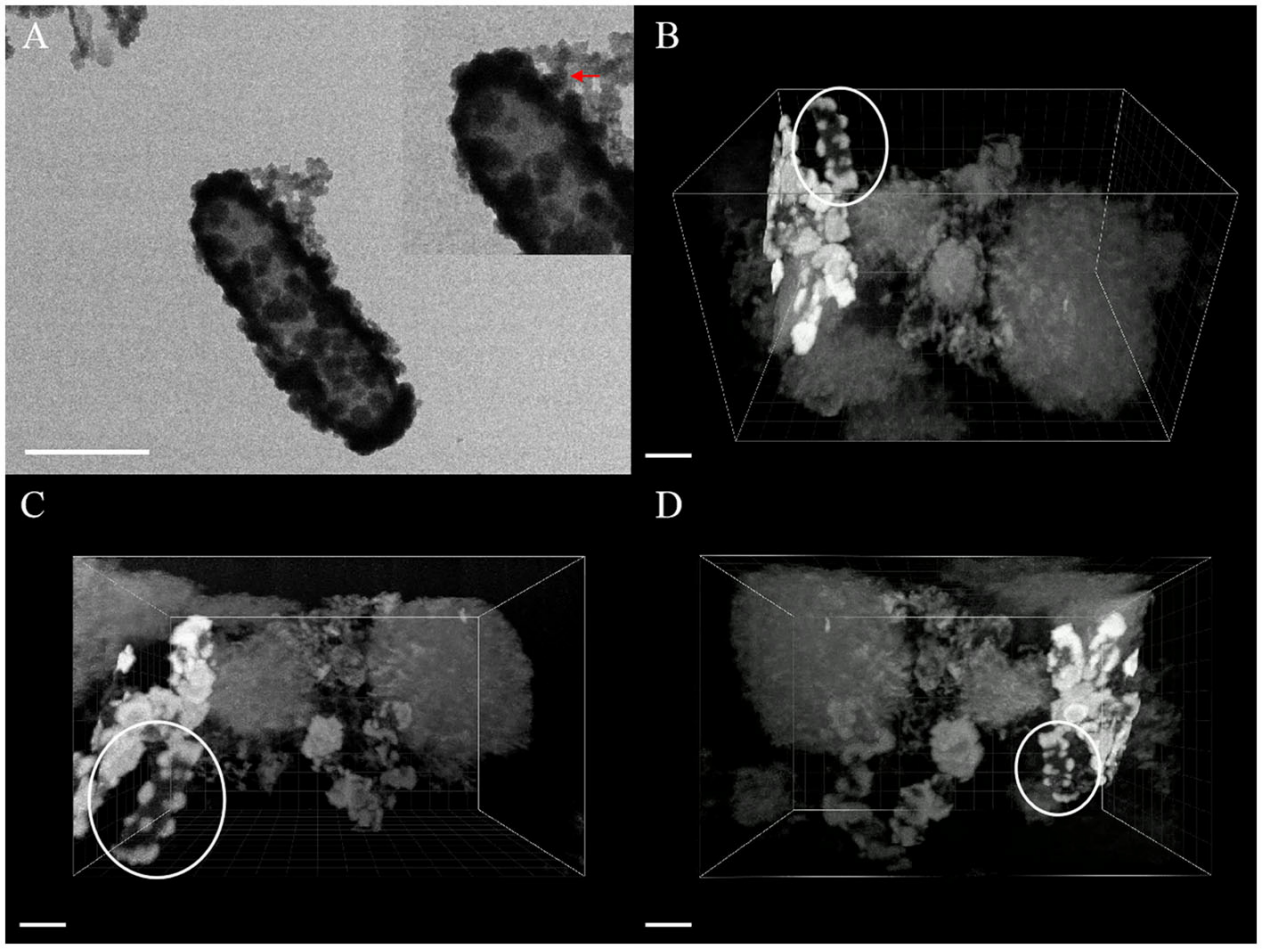
TEM image **(A)** and FIB/SEM serial images **(B–D)** of mineral globules [red arrow of inset in **(A)**, white circle in **(B–D)**] spread on cell surface, scale bar = 1 μm.

FIB–SEM 3D reconstruction enables us to better understand cell mineralization to avoid ineffective interpreting of 2D images. The morphology of cells in two stages and the morphology of extracellular minerals on the nano-sized scale were observed using the TEM images of cells of the same type to express the details of 3D imaging more clearly. After 3D imaging of samples of *Acidovorax* sp. strain BoFeN1 and combining with the TEM image results, we found that pH at the microscopic scale had no significant effect on 3D imaging, so we selected two representative 3D images to carry out the morphologic observations. The pH was 6.8 (**Figure 8**) and 6.9 (**Figure 7**).

As shown in the Figures 7, 8, it contained at least two completely crusted cells, and the compacted part was mainly cells. Referring to the same type of TEM diagram (Figure 8A), granules spread on the cell surface. In addition, large, loose, and massive aggregates were observed outside the cells (Figures 8B–D). XRD and Raman information showed that the product was goethite with low crystallinity, because the bulk aggregate was more dominant in the whole system. Bulky minerals were attached to the cell surface and interconnected with chain-like agglomerations of globular structures outside the cell (Schmid et al., 2014).

The composition of mineralized products of anaerobic iron-oxidizing bacteria depended on various factors, such as geochemical conditions, microbial community, and ferrous ion concentration (Bryce et al., 2018). The 3D reconstruction showed that the formation of minerals in the whole solution system went through the process of crystallization, dissolution, and recrystallization, from the minerals on the cell surface to the massive aggregates outside the cell. First, the minerals crystallized on the cell surface (see the circle in Figure 8), and then, due to the unique metabolic mechanism of ferric-oxidizing bacteria, the pH around the cell was reduced. At this time point, the mineralized products on the surface of the cell began to dissolve. The pH difference between the cell surface and the farther position away from the cell promoted their recrystallization at a distant position, resulting in the minerals transformed from disordered phase to more ordered crystals (see the large, loose, and massive aggregates in Figure 8) (Hegler et al., 2010). In this recrystallization, heavy metal ions can combine with mineral crystals and then precipitate in sediments, which promotes the immobilization of heavy metal ions (Abramov et al., 2020).

The recrystallization indicates that the mineralized products generated by bacterial metabolism on the cell surface have little influence on the composition of the final mineralized products. The results of Larese-Casanova consolidate the claim that *Acidovorax* sp. strain BoFeN1 has produced a lot of goethites and a small amount of lepidocrocite at pH 7.7, but produced almost all of the lepidocrocite at pH 6.3 (Larese-Casanova et al., 2010). Meanwhile, the bulk aggregates had a larger proportion in the whole system, which also confirmed that the geochemical conditions of the solution (including pH) were key factors that determined the final mineralized products of the anaerobic iron-oxidizing bacteria.

## Conclusion

The metabolic mechanisms for iron oxidation by *Acidovorax* sp. strain BoFeN1 and *Rhodobacter ferrooxidans* strain SW2 are different, and it is unsurprising that their mineralized products on the cell surface also differed. However, their metabolic activities respond similarly to changes in the environmental pH. It is found that at pH 6.8, the cell growth of *Rhodobacter ferrooxidans* strain SW2 and *Acidovorax* sp. strain BoFeN1 is inhibited and the crystallinity of goethite products is decreased. When pH is higher than 6.8, the mineralization process of the two strains can be improved, which may increase the possibility of adsorption and coprecipitation of heavy metal ions. The environmental pH can influence the metabolic activity of anaerobic iron-oxidizing bacteria, and in turn, both the two strains can reduce the pH around cells to localize minerals away from cells. Eventually, the pH of all culture solutions decreases to pH 5–6, which is the optimum pH range for the adsorption of many heavy metals. The 3D reconstruction shows that the mineralized products experience crystallization on the cell surface, dissolution, and recrystallization at distance from the cell. The observed massive aggregates in the system indicate that pH has a stronger influence on the final mineral structure of anaerobic iron-oxidizing bacteria than the oxidation processes. These minerals have the potential to adsorb and coprecipitate heavy metals. The higher crystallinity of the resulting minerals can make the heavy metal ions in the minerals more stable and less likely to be released. In this case, higher efficiency of bioremediation can be expected. Taken together, the abilities of *Rhodobacter ferrooxidans* strain SW2 and *Acidovorax* sp. strain BoFeN1 to adsorb heavy metal ions can be adjusted by slight changes in pH under neutrophilic conditions, and it has application prospects in bioremediation.

## Data Availability Statement

The raw data supporting the conclusions of this article will be made available by the authors, without undue reservation.

## Author Contributions

LH contributed to conception and design of the work. NJ, QH, and XL did the experiments. NJ, YG, and YF drew the illustrations. YF wrote the first draft of the manuscript. NJ, YF, ZY, CP, SL, and LH revised the manuscript. All authors read and approved the submitted version.

## Funding

This work was supported by grants from the National Natural Science Foundation of China (41877400), the National Key Research and Development Project of China (2018YFC1802601), the Startup Funding of the Chinese Academy of Sciences (2017-020), the State Key Laboratory of Environmental Geochemistry (SKLEG2018911), and the State Key Laboratory of Microbial Technology Foundation (M2017-01).

## Conflict of Interest

The authors declare that the research was conducted in the absence of any commercial or financial relationships that could be construed as a potential conflict of interest.

The handling editor declared a shared affiliation with the authors NJ, YF, QH, XL, YG, SL, and LH at the time of the review.

## Publisher's Note

All claims expressed in this article are solely those of the authors and do not necessarily represent those of their affiliated organizations, or those of the publisher, the editors and the reviewers. Any product that may be evaluated in this article, or claim that may be made by its manufacturer, is not guaranteed or endorsed by the publisher.
